# Multi-locus Genotypes Underlying Temperature Sensitivity in a Mutationally Induced Trait

**DOI:** 10.1371/journal.pgen.1005929

**Published:** 2016-03-18

**Authors:** Jonathan T. Lee, Matthew B. Taylor, Amy Shen, Ian M. Ehrenreich

**Affiliations:** Molecular and Computational Biology Section, Department of Biological Sciences, University of Southern California, Los Angeles, California, United States of America; New York University, UNITED STATES

## Abstract

Determining how genetic variation alters the expression of heritable phenotypes across conditions is important for agriculture, evolution, and medicine. Central to this problem is the concept of genotype-by-environment interaction (or ‘GxE’), which occurs when segregating genetic variation causes individuals to show different phenotypic responses to the environment. While many studies have sought to identify individual loci that contribute to GxE, obtaining a deeper understanding of this phenomenon may require defining how sets of loci collectively alter the relationship between genotype, environment, and phenotype. Here, we identify combinations of alleles at seven loci that control how a mutationally induced colony phenotype is expressed across a range of temperatures (21, 30, and 37°C) in a panel of yeast recombinants. We show that five predominant multi-locus genotypes involving the detected loci result in trait expression with varying degrees of temperature sensitivity. By comparing these genotypes and their patterns of trait expression across temperatures, we demonstrate that the involved alleles contribute to temperature sensitivity in different ways. While alleles of the transcription factor *MSS11* specify the potential temperatures at which the trait can occur, alleles at the other loci modify temperature sensitivity within the range established by *MSS11* in a genetic background- and/or temperature-dependent manner. Our results not only represent one of the first characterizations of GxE at the resolution of multi-locus genotypes, but also provide an example of the different roles that genetic variants can play in altering trait expression across conditions.

## Author Summary

Individuals’ phenotypes are often determined through interactions between their genotypes and the environment (or ‘GxE’). Despite substantial research on this topic, the underlying causes of GxE are not fully understood. This stems, in part, from the fact that most mapping studies focused on GxE do not define how combinations of loci collectively alter the relationship between genotype, environment, and phenotype. Here, we show that the temperature sensitivity of a yeast colony morphology trait is determined by five specific multi-locus genotypes involving seven environmentally responsive loci that segregate in a cross. Comparison of these genotypes enables us to determine how the phenotypic effects of the involved alleles change across genetic backgrounds and temperatures, and even suggests potential molecular mechanisms underlying GxE in our system. Thus, our findings illustrate how characterizing GxE at the resolution of causal multi-locus genotypes can provide rich insights into this phenomenon that might otherwise be difficult to obtain.

## Introduction

Genotype-by-environment interaction (or ‘GxE’) occurs when genetically distinct individuals exhibit different phenotypic responses to the environment [1,2]. Work to date suggests that GxE is an important contributor to heritable variation in many agriculturally, evolutionarily, and medically relevant phenotypes (as described in [3–6] and elsewhere). However, although ‘GxE’ has been extensively studied, there are few, if any, traits for which the underlying genetic basis of GxE is fully understood. This lack of detailed case studies may have a technical basis, as causal loci involved in GxE can act in an environment- and genetic background-dependent manner [7,8], making them difficult to detect. Improving understanding of GxE could therefore require characterizing how combinations of alleles, rather than individual loci, influence phenotype across environments.

**Fig 1:**
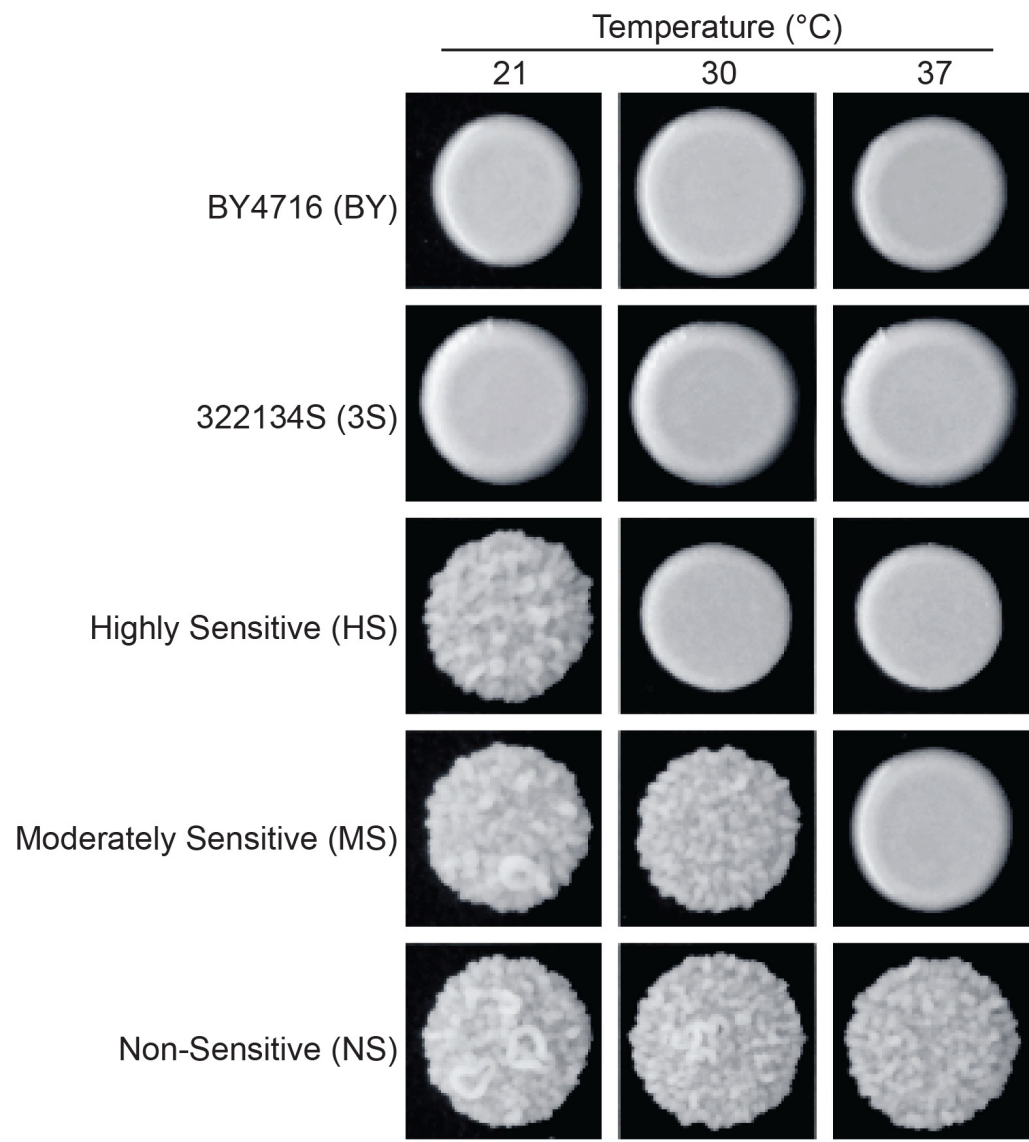
Segregants that carry *ira2Δ*2933 show different temperature sensitivities. BY4716 (BY), 322134S (3S), and their recombinant offspring typically exhibit ‘smooth’ colony morphology. However, in the presence of the *ira2Δ*2933 frameshift mutation, some BYx3S segregants are capable of expressing an alternative, ‘rough’ colony phenotype. Examination of rough BYx3S *ira2Δ*2933 segregants at 21, 30, and 37°C revealed that these individuals largely express the phenotype in a temperature sensitive manner. For the most part, individuals exhibit the phenotype only at 21°C, only at 21°C and 30°C, or at all examined temperatures. We refer to these three classes as highly sensitive (HS), moderately sensitive (MS), and non-sensitive (NS) to temperature, respectively.

We recently described a trait in *Saccharomyces cerevisiae* that can serve as a useful model for studying the complex genetic basis of GxE. In a cross of the BY4716 (‘BY’) lab strain and a derivative of the 322134S (‘3S’) clinical isolate [9], individuals typically exhibit ‘smooth’ colony morphology [10,11] (**Fig 1**). However, we showed that a spontaneous frameshift mutation in *IRA2*, a negative regulator of the Ras-cAMP-PKA (Ras) pathway [12,13], enables certain BYx3S segregants to express an alternative, ‘rough’ colony phenotype [10,11] (**Fig 1**). This mutation (*ira2*Δ2933) results in a truncation of the cognate Ira2 protein by 117 amino acids and causes a partial loss of Ira2 function [10]. However, *ira2*Δ2933 is insufficient to induce the rough phenotype on its own, as particular higher-order combinations of epistatically interacting alleles at the vesicle component *END3* [14,15], the transcriptional activators *FLO8* [16], *MGA1* [17,18], and *MSS11* [19], the transcriptional repressor *SFL1* [18,20], and the thioredoxin reductase *TRR1* [21] are also needed [10,11,22]. Specifically, we identified two multi-locus genotypes—*END3*^BY^ *FLO8*^3S^ *ira2*Δ2933 *MSS11*^BY^ *TRR1*^3S^ and *END3*^3S^ *FLO8*^3S^ *ira2*Δ2933 *MGA1*^BY^ *MSS11*^BY^ *SFL1*^BY^—that can cause the rough phenotype.

The aforementioned results stem from work performed exclusively at 30°C, the standard temperature used to culture *S*. *cerevisiae* in the lab. Here, we extend our research on the rough phenotype to two additional temperatures: 21°C and 37°C. In doing so, we find that many BYx3S *ira2Δ*2933 segregants express the rough phenotype in a temperature sensitive manner (**Fig 1**). To determine the genetic basis of this GxE, we perform genetic mapping of several temperature sensitivity classes using a combination of bulk segregant analysis [23–25] and selective genotyping of individual cross progeny [10,26]. These efforts lead to the identification of seven environmentally responsive loci, and five specific multi-locus genotypes involving these loci, that influence the expression of the rough phenotype across temperatures. As we describe below, comparison of these multi-locus genotypes provides detailed insights into the genetic architecture of temperature-dependent GxE in our system, and also sheds light on the distinct roles that the causal alleles play in modifying the rough phenotype’s expression at different temperatures.

## Results and Discussion

### Screen for temperature sensitivity among rough BYx3S *ira2*Δ2933 segregants

We screened for rough BYx3S *ira2*Δ2933 segregants at three temperatures—21, 30, and 37°C—in a backcross of the rough *ira2*Δ2933 segregant described in [10] to BY (S1 and S2 Tables; Materials and Methods). ~3,000 segregants (30 random spore plates of ~100 colonies per plate) were examined at each temperature and ~9,000 segregants were screened in total across the three temperatures (Materials and Methods). Among the 252 rough individuals obtained from this screen, 173, 107, and 72 were recovered from 21, 30, and 37°C, respectively (**S1** and **S2 Tables**). The majority of these rough segregants were capable of expressing the phenotype at temperatures other than the one from which they were collected. When these rough individuals were individually examined at each of the three initially employed temperatures, they largely fell into three classes: rough at 21°C only, rough at 21 and 30°C only, or rough at all three temperatures (Fig 1; S1 and S3 Tables; Materials and Methods). This implies that the major form of temperature-dependent GxE in our system is temperature sensitivity. For the remainder of the paper, we refer to the three aforementioned classes as highly sensitive (‘HS’), moderately sensitive (‘MS’), and non-sensitive (‘NS’) to temperature, respectively (**Fig 1**).

We sought to determine the underlying genetic basis of the temperature-dependent GxE by selectively genotyping individuals in each temperature sensitivity class. To generate the necessary populations for this genetic mapping strategy, we screened an additional 60 plates of random spores from the BY backcross mentioned above, and also mated the rough *ira2*Δ2933 segregant described in [10] to its 3S parent and screened 90 random spore plates from this 3S backcross (Materials and Methods). Given that spores were plated at a density of ~100 colonies per plate, we estimate that we examined an additional ~15,000 individuals as a part of this second screen. This second screen was exclusively conducted at 21°C, as all three temperature sensitivity classes can be recovered from this condition (**S2 Table**). Collected individuals were then stringently phenotyped at 21, 30, and 37°C (Materials and Methods). In combination with our preliminary screen, we recovered 544 and 466 rough backcross progeny from the BY and 3S backcrosses, respectively, with 78.4% of these individuals classified as HS, MS, or NS (S1 and S4 Tables; Materials and Methods).

### Bulk segregant mapping of the three temperature sensitivity classes

**Fig 2:**
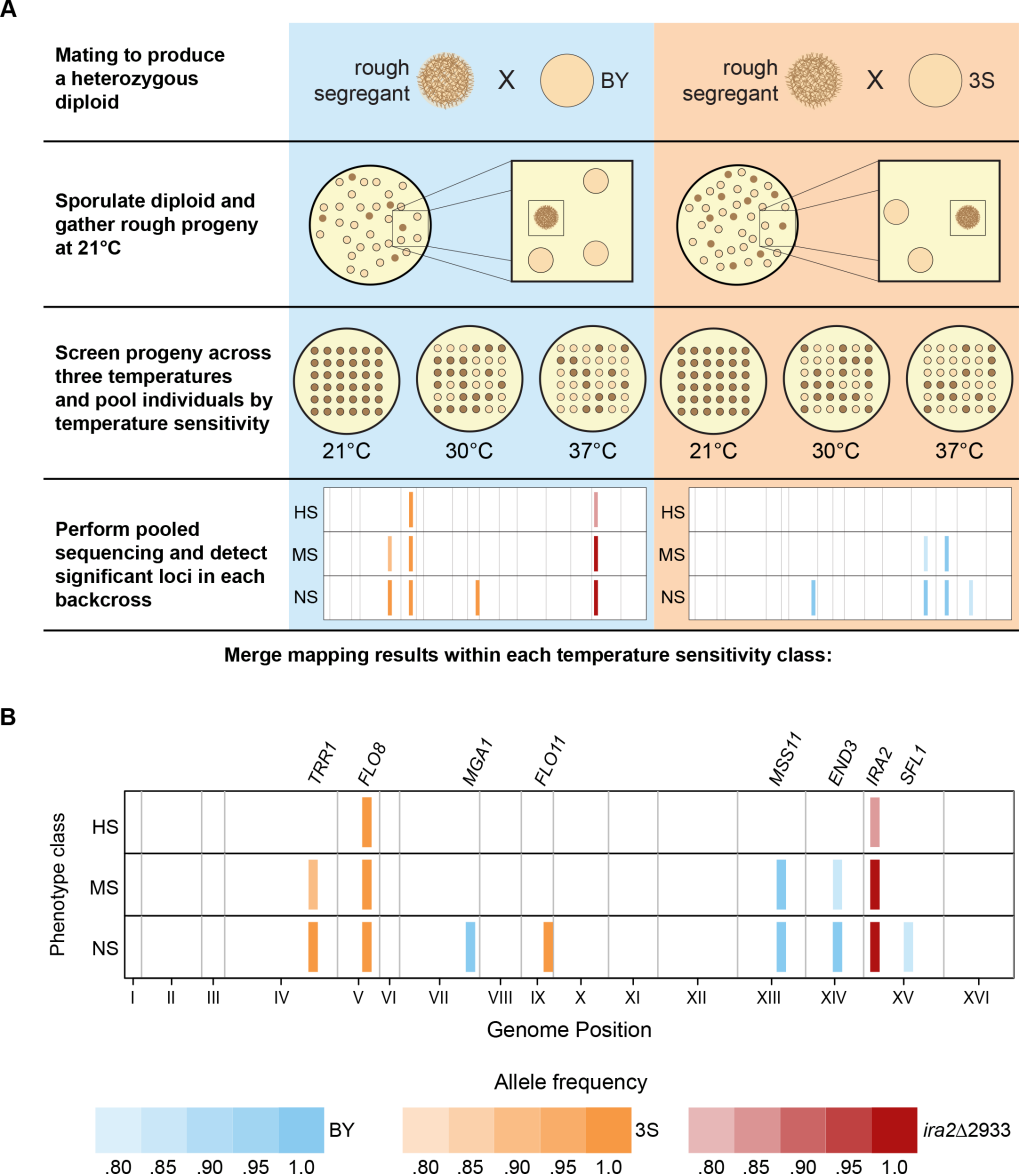
Bulk segregant mapping results for different temperature sensitivity classes. (**A**) A rough BYx3S *ira2Δ*2933 F_2_ segregant was backcrossed to the BY and 3S strains. Progeny from both of these backcrosses were screened at 21, 30, and 37°C to identify individuals in each temperature sensitivity class. Bulk segregant mapping was then performed on each temperature sensitivity class in each backcross. (**B**) Eight total loci were detected, seven of which we had previously resolved to specific genes and one of which we cloned in the current paper. The identities of these genes are stated above their corresponding loci. Alleles detected from BY and 3S are shown in blue and orange, respectively, while the *ira2*Δ2933 mutation is denoted in red. The color intensity of a locus corresponds to its allele frequency in the bulk segregant mapping data. A legend with the correspondence between allele frequency and color intensity is provided at the bottom of the figure. To aid in visualization, loci are depicted in the main text figures as having the same widths.

**Fig 3:**
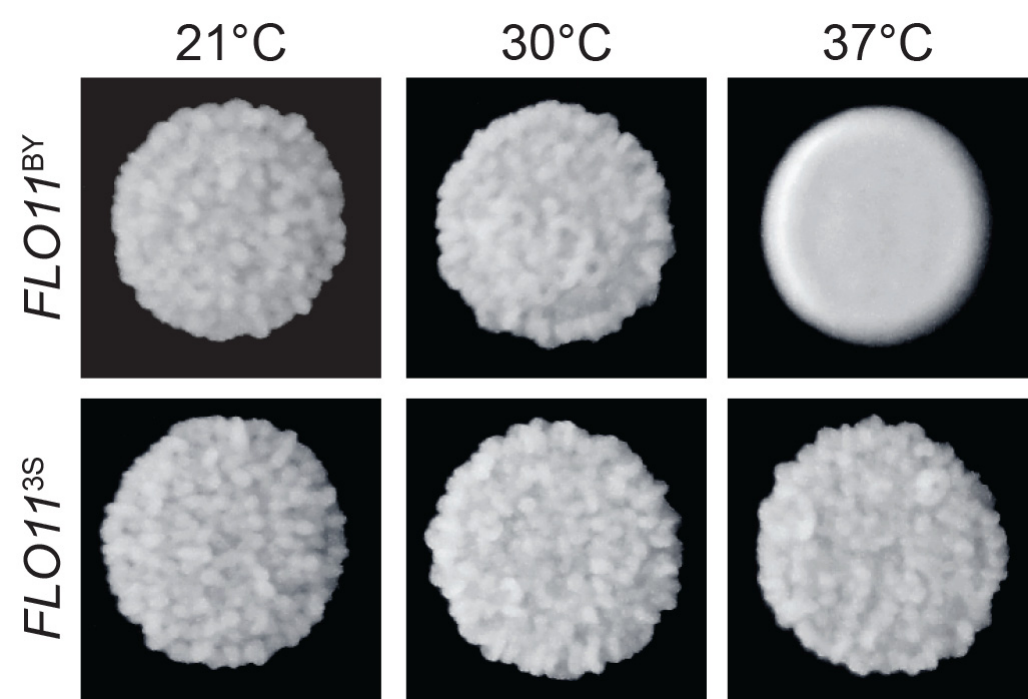
*FLO11*^3S^ is required for trait expression at 37°C. We replaced *FLO11*^3S^ with *FLO11*^BY^ in a rough BY backcross segregant from the NS class with the genotype *END3*^BY^ *FLO8*^3S^ *FLO11*^3S^ *ira2*Δ2933 *MGA1*^BY^ *MSS11*^BY^ *SFL1*^BY^ *TRR1*^3S^. This allele replacement resulted in a conversion from rough to smooth colony morphology specifically at 37°C. In this picture, the *FLO11*^3S^ individual is a segregant that has not been genetically modified, while the *FLO11*^BY^ individual is the same strain with its *FLO11* allele swapped. The phenotypes of both of these strains at 21, 30, and 37°C are shown.

We first attempted to determine the genetic bases of the three temperature sensitivity classes using bulk segregant mapping by sequencing [23–25] (**Fig 2A**). Between 51 and 126 individuals were pooled per backcross and temperature sensitivity class, and each pool was sequenced to at least 114X coverage (S4 Table; Materials and Methods). Across the six pools, eight distinct loci were detected using MULTIPOOL [27] (Figs 2B and S1; Materials and Methods). Seven of these loci overlapped *ira2Δ*2933 or causal alleles that we previously identified as contributors to the phenotype at 30°C: *END3*^BY^, *FLO8*^3S^, *MGA1*^BY^, *MSS11*^BY^, *SFL1*^BY^, and *TRR1*^3S^ [10,11] (**Fig 2B**). We used genetic engineering to show that the final locus, which was detected on Chromosome IX in the NS class, corresponds to the 3S allele of *FLO11*, which encodes a cell surface glycoprotein that is required for the rough phenotype [11] (Materials and Methods). Specifically, we replaced the 3S version of the *FLO11* coding region with the BY allele in an NS individual from the BY backcross, and found the resulting allele swap strain only exhibited rough morphology at 21 and 30°C (**Fig 3**).

Loci that contributed to temperature sensitivity could be distinguished from those that did not based on the bulk segregant mapping results. *FLO8*^3S^ and *ira2Δ*2933 were detected in every temperature sensitivity class (**Fig 2B**; **S1 Note**), suggesting they are involved in the general expression of the rough phenotype but do not influence its temperature sensitivity. In contrast, the other six involved alleles—*END3*^BY^, *FLO11*^3S^, *MGA1*^BY^, *MSS11*^BY^, *SFL1*^BY^, and *TRR1*^3S^—were each detected in just one or two of the classes, indicating they contribute to the observed differences in temperature sensitivity (**Fig 2B**). Of these six alleles, zero, three, and six were detected in the HS, MS, and NS classes, respectively, and the three that were detected among MS individuals—*END3*^BY^, *MSS11*^BY^, and *TRR1*^3S^—were also identified among NS individuals. These findings indicate that the temperature sensitivity of the rough phenotype is largely controlled by the same loci that were originally determined to underlie the trait’s expression at 30°C [10,11], and that the rough phenotype’s temperature sensitivity can be reduced or even eliminated by combining particular alleles at these loci. In particular, we note that individuals with the genotype *END3*^BY^ *FLO8*^3S^ *FLO11*^3S^ *ira2Δ*2933 *MGA1*^BY^ *MSS11*^BY^ *SFL1*^BY^ *TRR1*^3S^ did not exhibit any temperature sensitivity in our experiments.

### Multiple genotypes underlie the HS and MS classes

**Fig 4:**
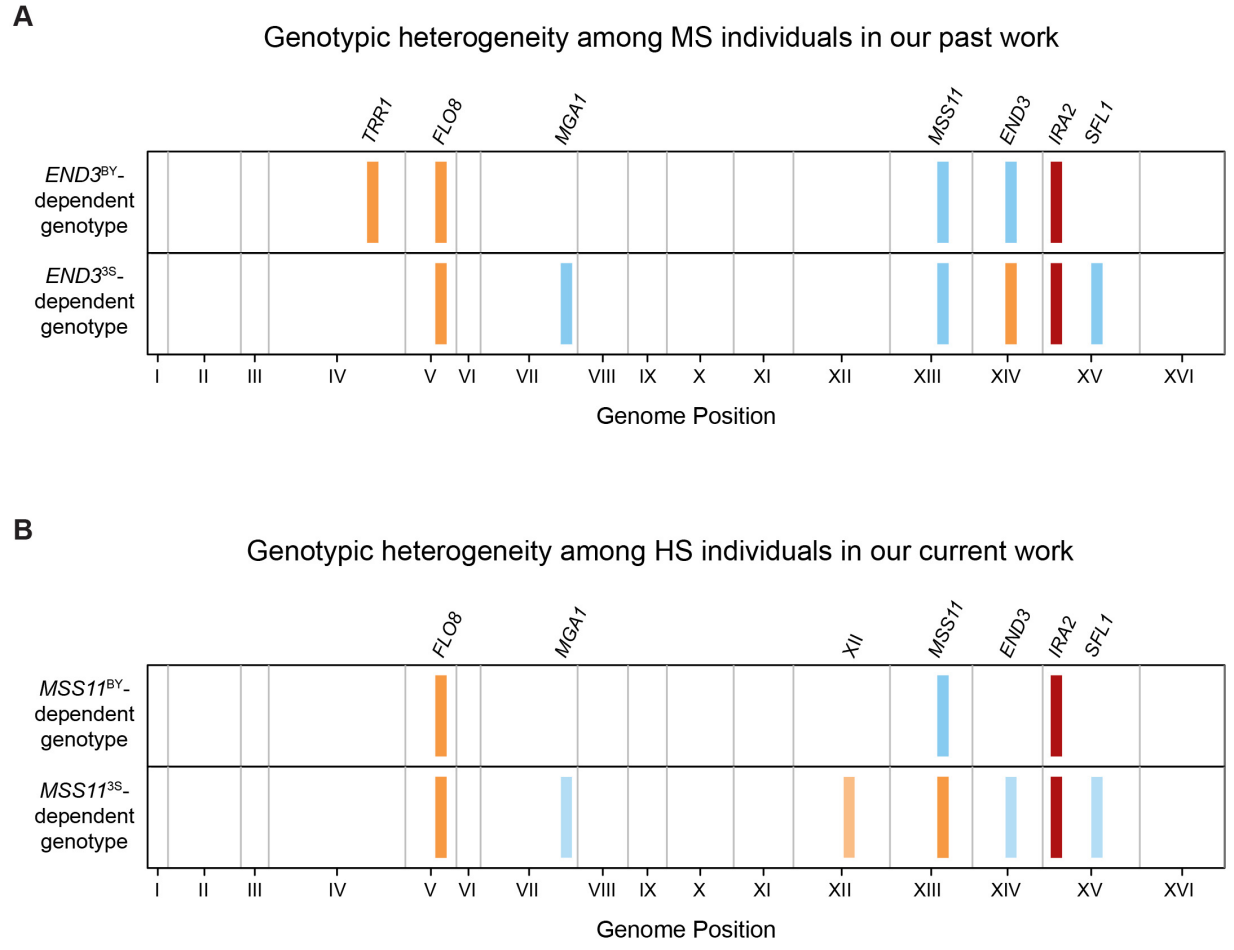
The HS and MS classes are each specified by two predominant multi-locus genotypes. (**A**) We previously showed that two distinct multi-locus genotypes underlie trait expression at 30°C [10,11], and in this paper we determined that these same genotypes underlie the MS class. In addition to *ira2Δ*2933, these genotypes both involve *FLO8*^3S^ and *MSS11*^BY^. However, individuals carrying *END3*^BY^ also require *TRR1*^3S^ (‘*END3*^BY^-dependent genotype’), while individuals carrying *END3*^3S^ instead require *MGA1*^BY^ and *SFL1*^BY^ (‘*END3*^3S^-dependent genotype’). We refer to the fact that both of these multi-locus genotypes specify the same trait as ‘genotypic heterogeneity’. When such genotypic heterogeneity is present, alleles involved in only one of the multi-locus genotypes can be masked. For example, in the bulk segregant mapping data for the MS class, only the alleles involved in the genotype that involves *END3*^BY^ were detected. (**B**) In the current data, we found evidence for genotypic heterogeneity in the HS class. By partitioning individuals in the HS class from the 3S backcross population and calculating their allele frequencies across the genome, we determined that two predominant multi-locus genotypes underlie this temperature sensitivity class. These genotypes are *FLO8*^3S^ *ira2Δ*2933 *MSS11*^BY^ (‘*MSS11*^BY^-dependent genotype’) and XII^3S^ *END3*^BY^ *FLO8*^3S^ *ira2Δ*2933 *MGA1*^BY^ *MSS11*^3S^ *SFL1*^BY^ (‘*MSS11*^3S^-dependent genotype’). As described in the main text, XII^3S^ refers to a locus that was detected specifically among HS individuals carrying *MSS11*^3S^. The same coloring scheme is used in this figure as in **Fig 2**.

Although bulk segregant mapping is known to be a statistically powerful technique when large numbers of cross progeny are examined [24], it can fail to detect causal loci if distinct combinations of alleles that interact with each other or the environment exhibit indistinguishable phenotypes [10,26]. This phenomenon, which we refer to here as ‘genotypic heterogeneity’, clearly occurred in the present data for the MS class. As described in the introduction, we previously showed that two specific multi-locus genotypes—*END3*^BY^ *FLO8*^3S^ *ira2*Δ2933 *MSS11*^BY^ *TRR1*^3S^ and *END3*^3S^ *FLO8*^3S^ *ira2*Δ2933 *MGA1*^BY^ *MSS11*^BY^ *SFL1*^BY^—express the rough phenotype at 30°C [11] (**Fig 4A**). Phenotyping of previously described segregants [10,11] revealed that both of these allele combinations lead to moderate temperature sensitivity—i.e., expression of the trait at 21 and 30°C, but not 37°C (**S2 Fig**). In our current data for the MS class, alleles required for both genotypes were fixed, while alleles involved in just one of the two genotypes were merely enriched or not even detected (**Fig 2B**; **S2 Note**). To directly show that our sample of MS individuals was comprised of both previously identified multi-locus genotypes, we genotyped 19 random MS segregants using restriction markers for *END3* and *MGA1*. This effort revealed that 14 and 5 of these individuals possessed the *END3*^BY^ *FLO8*^3S^ *ira2*Δ2933 *MSS11*^BY^ *TRR1*^3S^ and *END3*^3S^ *FLO8*^3S^ *ira2*Δ2933 *MGA1*^BY^ *MSS11*^BY^ *SFL1*^BY^ genotypes, respectively (S3 Note; Materials and Methods).

After recognizing the genotypic heterogeneity underlying the MS class (**Fig 4A**), we investigated whether the HS class, for which only *FLO8*^3S^ and *ira2Δ*2933 were detected by bulk segregant mapping (**Fig 2A**), might also be genotypically heterogeneous. To examine this possibility, we individually genotyped each HS segregant by performing low coverage whole genome sequencing (Materials and Methods). We then used χ^2^ tests to scan the genomes of the *ira2Δ*2933 segregants in the HS class for pairs of loci that exhibited correlated allele states (Materials and Methods). Such associations might be expected if alleles at two loci participate in the same multi-locus genotype. At a 1% false discovery rate threshold, we detected no significant pairs of loci in the BY backcross and two significant pairs of loci in the 3S backcross: Chromosome XII-Chromosome XIII and Chromosome XIII-Chromosome XV (S3 Fig; Materials and Methods). Both of these significant locus pairs included a region of Chromosome XIII that overlapped *MSS11*. As for the other two detected loci, the Chromosome XV region overlapped *SFL1* and the Chromosome XII region was novel relative to our past work [10,11]. As we have yet to determine the causal gene at the Chromosomes XII locus (**S4 Note**), we hereafter refer to it by its chromosome number: ‘XII’.

Detection of correlated loci among individuals from the 3S backcross suggested that genotypic heterogeneity might have led to only *FLO8*^3S^ and *ira2Δ*2933 being identified by bulk segregant mapping focused on the HS class (**Fig 2B**). Because *MSS11* was present in both significant locus pairs, we split the 3S backcross progeny by individuals’ genotypes at *MSS11* and separately examined genome-wide allele frequencies in the two resulting groups (Materials and Methods). This analysis revealed that individuals with *MSS11*^BY^ only need the specific alleles *ira2Δ*2933 and *FLO8*^3S^ to express the phenotype at 21°C, whereas individuals with *MSS11*^3S^ require a number of additional alleles to show rough morphology under the same condition (Fig 4B; Materials and Methods). Specifically, XII^3S^, *END3*^BY^, *MGA1*^BY^, and *SFL1*^BY^ collectively enable *FLO8*^3S^ *ira2Δ*2933 *MSS11*^3S^ individuals to express the trait at 21°C (Fig 4B; Materials and Methods). We validated this finding by performing allele replacements of *END3*, *MGA1*, and *SFL1* in a rough segregant from the 3S backcross that possessed the XII^3S^ *END3*^BY^ *FLO8*^3S^ *ira2Δ*2933 *MGA1*^BY^ *MSS11*^3S^ *SFL1*^BY^ genotype (Materials and Methods). These replacements each resulted in the engineered strain being incapable of expressing the rough phenotype at 21°C, implying that the detected alleles have biologically meaningful effects on the trait in the HS background involving *MSS11*^3S^ (**S4 Fig**). Thus, two predominant genotypes underlie the HS class: *FLO8*^3S^ *ira2Δ*2933 *MSS11*^BY^ and XII^3S^ *END3*^BY^ *FLO8*^3S^ *ira2Δ*2933 *MGA1*^BY^ *MSS11*^3S^ *SFL1*^BY^.

### Roles of detected alleles in modulating temperature sensitivity

**Fig 5:**
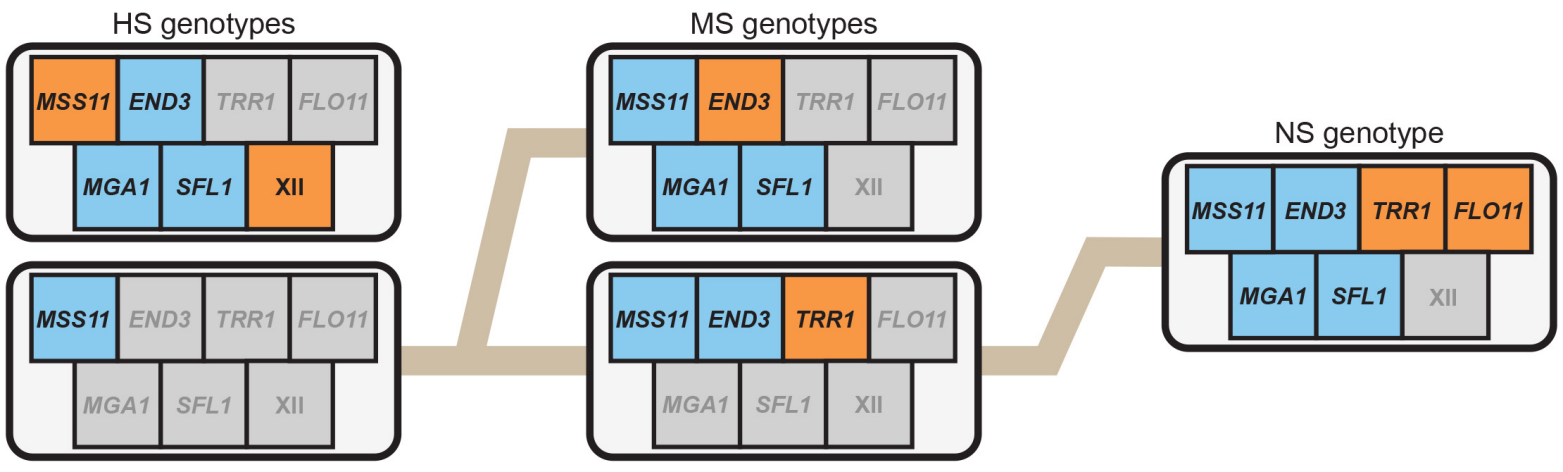
Comparison of multi-locus genotypes underlying differences in temperature sensitivity among BYx3S *FLO8*^3S^ *ira2Δ*2933 segregants. Across our current study and past work, we have identified five predominant multi-locus genotypes that underlie the three temperature sensitivity classes. Excluding *FLO8*^3S^, which is generally required for expression of the trait in the presence of *ira2Δ*2933, these genotypes involve specific alleles at seven environmentally responsive loci. Respectively, blue and orange indicate the BY or 3S allele of a given gene participates in a specific multi-locus genotype, while grey indicates that neither allele is required. Additionally, brown lines emphasize how adding particular combinations of alleles to a *FLO8*^3S^ *ira2Δ*2933 *MSS11*^BY^ genetic background can lead to reduction or elimination of temperature sensitivity.

Based on our genetic mapping efforts described in this paper, we have identified seven environmentally responsive loci that influence the expression of the rough phenotype across temperatures: XII, *END3*, *FLO11*, *MGA1*, *MSS11*, *SFL1*, and *TRR1*. Furthermore, through our current and past efforts [10,11], we have characterized five predominant multi-locus genotypes involving these loci that exhibit different levels of temperature sensitivity. These include two HS genotypes, two MS genotypes, and a single NS genotype (**Fig 5**).

Comparison of the alleles involved in these five multi-locus genotypes suggests that the seven environmentally responsive loci play different roles in modifying the rough phenotype’s temperature sensitivity. In particular, *MSS11* appears to determine the range of temperatures at which the phenotype can be expressed (**Fig 5**). Indeed, while both *MSS11* alleles can facilitate expression of rough morphology at 21°C, only individuals that carry *MSS11*^BY^ have the genetic potential to express the trait at 30 or 37°C (**Fig 5**). In contrast, the other identified alleles appear to collectively increase or decrease temperature sensitivity within the range established by *MSS11* (**Fig 5**). These modifier alleles show phenotypic effects that can depend on both genetic background and temperature, although the degree of such dependencies varies. For example, *END3*^BY^, *MGA1*^BY^, and *SFL1*^BY^ were detected in one multi-locus genotype in each temperature sensitivity class (**Fig 5**), and appear to influence temperature sensitivity in a genetic background-dependent manner (see **S4** and **S5 Figs**, as well as [10], for genetic engineering results supporting this point for *MGA1*^BY^). The remaining alleles appear to have effects on temperature sensitivity that depend on both genetic background and temperature. Specifically, XII^3S^ and *FLO11*^3S^ were each detected in a single multi-locus genotype and temperature sensitivity class, while *TRR1*^3S^ was only identified in the *END3*^BY^ *MSS11*^BY^ genetic background among MS and NS individuals (**Fig 5**).

### Conclusion

Across the current manuscript and our previous work [10,11], we have now described eight loci and five predominant multi-locus genotypes that influence whether BYx3S segregants carrying *ira2Δ*2933 can express the rough phenotype in at least one temperature. Of the eight identified loci, only one—the transcription factor *FLO8*—does not contribute to temperature sensitivity. This likely reflects the fact that *FLO8* encodes a transcriptional activator that is required for expression of the rough phenotype [10], and BY harbors a nonfunctional version of this gene [26,28]. For the remaining seven loci, it is difficult to distinguish between their effects on trait expression in general and their effects on temperature sensitivity. Indeed, our results indicate that genetic background and temperature together determine which alleles are required for expression of the rough phenotype.

Our findings also suggest molecular mechanisms that might underlie the rough phenotype’s temperature sensitivity. For instance, some of the alleles involved in temperature sensitivity may have reduced biochemical activity and/or diminished structural stability at 30 or 37°C. The most striking example of this is *MSS11*^3S^, which can only facilitate trait expression at 21°C. Given that Mss11 acts as a heterodimer with Flo8 [29], it might be that Flo8-Mss11^3S^ does not dimerize well, poorly binds DNA, or is unable to stimulate RNA polymerase II activity at 30 and 37°C. Supporting such possibilities, we determined that the causal variant in *MSS11* is an isoleucine to serine amino acid change that occurs in the LisH motif required for dimerization with Flo8 [29] (**S6 Fig**). 3S and roughly half of the other available sequenced *S*. *cerevisiae* isolates carry the derived version of this site, which is the serine allele that results in temperature sensitivity (**S6 Fig**). The data also suggest *FLO11*, which plays a crucial role in cell-cell and cell-surface adhesion [30,31], harbors a temperature sensitive polymorphism in its coding region. Instability of Flo11^BY^ at 37°C could result in temperature-dependent suppression of the rough phenotype among *FLO11*^BY^ individuals and would explain why *FLO11*^3S^ is required by the NS class.

Temperature sensitivity at the phenotypic level may also be caused by the combined effects of genetic background and temperature on Ras signaling and Ras-dependent gene regulation. We note that Flo8-Mss11, Mga1, and Sfl1 are all Ras-regulated transcription factors [18]. Furthermore, Flo8-Mss11 and Sfl1 are known to play particularly important roles in the expression of multicellular phenotypes in yeast, as they compete to bind DNA and are regulated in an antagonistic manner at the posttranslational level by Protein Kinase A, the effector kinase of the Ras cascade [30,32,33]. In fact, we previously showed that *ira2Δ*2933 reveals the rough phenotype in certain genetic backgrounds by reducing Sfl1-mediated transcriptional repression [11]. Viewing our current work in light of our past findings suggests that the temperature sensitivity described in this paper results in part from genotype-temperature combinations that conditionally reduce Ras signaling and/or Ras-regulated gene expression. Consistent with this possibility, others have noted functional relationships between Ras signaling and both End3 [34] and the oxidative stress response, of which Trr1 is a component, in yeast [34,35]. Additionally, temperature has been reported to affect levels of Ras signaling in human fibroblast cultures [36].

Our results also shed light on the genetic basis of phenotypic capacitance—i.e., the uncovering of cryptic genetic variation by environmental or mutational perturbation [37–42]. As we previously described, all of the polymorphisms that influence rough morphology in the BYx3S *ira2Δ*2933 cross can be considered cryptic genetic variants, as they do not cause the rough phenotype under standard conditions in the absence of the *IRA2* mutation [11]. Here, we have shown the multi-locus genotypes that provide the genetic potential for *ira2*Δ2933 to uncover the rough phenotype differ across temperatures. Moreover, we have demonstrated that these multi-locus genotypes that facilitate phenotypic capacitance vary not only in their initial temperature sensitivities, but also in their potential to reduce their temperature sensitivities through segregating genetic variation (**Fig 5**). This latter finding has potential relevance for our understanding of genetic assimilation, the process by which environmentally induced traits are converted into constitutively expressed phenotypes by natural selection [42–56], as our results provide an example of the genetic architecture that might underlie this phenomenon.

Lastly, our study provides technical insights into research aimed at determining the genetic basis of GxE. First, we have shown that selective genotyping of individuals that exhibit particular levels of environmental sensitivity can identify multi-locus genotypes that cause GxE, rather than just individual contributing loci. Second, we have demonstrated that genotypic heterogeneity can complicate efforts to genetically dissect GxE and have described a strategy to overcome this challenge. Third, we have illustrated how characterizing the genetic basis of GxE at the resolution of multi-locus genotypes can clarify the different roles that contributing loci play in altering trait expression across conditions. These technical insights that have emerged from our work will likely be relevant to future studies of GxE in other species and traits.

## Materials and Methods

### Generation of backcross segregants for the preliminary screen and genetic mapping

The rough BYx3S *MAT****a*** F_2_ segregant used for genetic mapping in [10] was mated to *MATα* versions of both BY and 3S. Diploid zygotes were obtained from each backcross mating using microdissection, and then sporulated at 21°C using standard yeast sporulation methods [57]. Spore cultures were digested with β-glucoronidase and random *MAT****a*** spores were selected on yeast nitrogen base (YNB) plates containing canavanine using the Synthetic Genetic Array (SGA) marker system [58], as described previously [10,24]. Implementation of the SGA system was possible because the *MAT****a*** F_2_ segregant used in backcrossing possessed the markers *can1*Δ::*STE2pr-SpHIS5* and *his3*Δ [58], and matings were performed to BY and 3S *MATα his3Δ* strains. Spores were plated at low density (~100 spores per plate) so that individual colonies could be easily distinguished. After five days of growth on YNB + canavanine plates, colonies were replicated onto yeast extract-peptone-ethanol (YPE) plates. These YPE plates were incubated at the specified temperature (21, 30, or 37°C) for five days and then screened by eye for colonies exhibiting the rough phenotype. Strains identified as rough were picked from the plates, inoculated in liquid yeast extract-peptone-dextrose (YPD) media, and grown overnight at 30°C. Freezer stocks of the rough backcross segregants were generated by mixing equal volumes of 40% glycerol solution with a portion of the liquid YPD cultures and then storing these cultures at -80°C.

### Phenotyping of rough segregants at multiple temperatures

Cells from the freezer stocks described above were inoculated into 800 μl of liquid YPD media. These YPD cultures were grown for two days at 30°C and then manually pinned onto three YPE plates. Of these three plates, one was incubated at 21°C, one was incubated at 30°C, and one was incubated at 37°C. Individuals were scored manually on a scale of 0 to 5 based on their degree of expression of the rough phenotype. The low and high ends of this scale correspond to individuals that were smooth and rough, respectively. Individuals with intermediate scores were bumpy to varying degrees, which may reflect weaker expression of the phenotype. Three biological replicate cultures were screened for each segregant. For the purposes of the paper, we viewed an individual’s phenotype at a given temperature as their median score obtained for that temperature. The results described in the paper are based on individuals that clearly and consistently showed smooth or rough phenotypes at a given temperature. We considered median scores between 0 and 1 or 4 and 5 as clearly indicating the smooth and rough phenotypes, respectively. Phenotype data are reported in **S1 Table**.

### Bulk segregant mapping of temperature sensitivity

Cultures were inoculated from the freezer stocks described above into 800 μl of liquid YPD. After two days of growth at 30°C, 100 μl of each culture from the same backcross and temperature sensitivity class were mixed together, and DNA was extracted from these pools using the Qiagen DNeasy Blood and Tissue kit. DNA sequencing libraries were then constructed using the Illumina Nextera kit. Each library was barcoded with a distinct sequence tag to facilitate multiplex sequencing. Libraries were mixed in equimolar fractions and sequenced on an Illumina NextSeq machine using 75x75 or 150x150 base pair reads. Sequencing reads were mapped to both the reference genome for S288c, which is the progenitor of BY, and the 3S draft genome (available through http://www.yeastgenome.org) using the Burrows-Wheeler Aligner (BWA) version 7 with options mem -t 20 [59]. We then used SAMtools to obtain mpileup files for each sample [60]. Based on these mpileups, we determined that the pools were sequenced to an average per site coverage of at least ~114X (**S4 Table**). Genome-wide allele frequencies were determined at 36,756 high confidence SNPs that were previously identified by mapping Illumina sequencing reads for 3S to the S288c reference genome [10,11]. Because roughly half of the genome is fixed in each backcross, we subsetted out the data for segregating regions in each backcross and analyzed each of these regions individually. To identify significantly enriched loci, we used MULTIPOOL [27] with the settings: replicates mode, 3,300 bp centimorgans, 100 bp bins. Significant loci were defined as genomic regions that had a maximum LOD score of at least 5 for a span of at least 20 kb. Confidence intervals were estimated as the bounds of a locus that correspond to a 2 LOD drop from the point of maximal significance. To generate allele frequency plots, the data was smoothed by averaging allele frequency over sliding windows of 25 SNPs.

### Genotyping of MS individuals using PCR and restriction digestion

DNA was extracted from 19 randomly chosen BY backcross segregants from the MS class using the Qiagen DNeasy Blood and Tissue kit. Small regions of these genes that contained a SNP were amplified by PCR and then digested with restriction enzymes (see **S5 Table** for a description of specific reagents). The amplified regions were chosen so that one parental allele would be cut a single time while the other parental allele would not be cut at all. Each diagnostic restriction digest was tested on BY and 3S. Digested PCR products were examined on a 1.5% agarose gel containing ethidium bromide to determine each individual’s genotype at a given locus.

### Detailed genetic characterization of the HS class

Respectively, 68 and 89 HS segregants were independently prepared for sequencing from the backcrosses to BY and 3S. Individual cultures were generated by inoculating 800 μl of liquid YPD with cells from the freezer stocks described above. After two days of growth at 30°C, DNA was extracted from each individual culture using the Qiagen DNeasy 96 Blood and Tissue kit. Libraries were prepared for sequencing using the Illumina Nextera kit, with each individual receiving a unique sequence barcode. Sequencing was performed on an Illumina NextSeq machine using 75x75 base pair reads. Illumina reads were then mapped to the S288c genome, and mpileups were generated from these alignments using BWA [59] and SAMtools [60] in the same manner described earlier for the bulk segregant mapping data. Individuals with an average per site coverage below 1.02X were excluded from subsequent analyses. Respectively, one and five individuals were excluded from the BY and 3S backcrosses due to low coverage. Furthermore, 16 sequenced individuals from the BY backcross were excluded from downstream analysis because they possessed a wild type *IRA2* (**S1 Note**). As previously described [10], a Hidden Markov Model (HMM) was used to determine the haplotype of each segregant from the aforementioned 36,756 SNP differences between the BY and 3S genomes. As with the bulk segregant data, we only performed our statistical tests on regions of the genome that segregated in a given backcross. Furthermore, to reduce our total number of statistical tests, we collapsed linked SNPs that showed the same pattern of inheritance across backcross segregants into unique segregating regions. There were 1,399 and 767 such segregating regions in the BY and 3S backcross populations, respectively. We used the R statistical programming environment to identify pairs of loci on different chromosomes that showed correlated alleles states based on *Χ*^2^ tests. Pairs of loci were considered statistically significant if they exhibited a point-wise false discovery rate (q-value) of 1% or less, as determined when p-values for all segregating regions were converted into q-values by the qvalue() package in R [61,62]. Regions within 20,000 bases of the ends of chromosomes were excluded from this analysis due to the problems in mapping Illumina reads to telomeric regions. Based on the results from the initial scan for correlated loci, HS individuals from the 3S backcross were subsetted by their genotype at *MSS11* and genome-wide allele frequency plots were generated from the aforementioned HMM tables. Allele frequencies were averaged over 25 SNP sliding windows, and loci were called as significant in a given *MSS11* background if they exhibited allele frequencies below 10% or greater than 90%.

### Genetic engineering

Adaptamer-mediated allele replacement [26,63] was used to alter the allele state at *MGA1* and *FLO11* in a NS segregant with the genotype *END3*^BY^ *FLO8*^3S^ *FLO11*^3S^ *ira2Δ*2933 *MGA1*^BY^ *MSS11*^BY^ *SFL1*^BY^ *TRR1*^3S^, as well as *END3*, *MGA1*, and *SFL1* in a HS segregant with the genotype XII^3S^ *END3*^BY^ *FLO8*^3S^ *ira2Δ*2933 *MGA1*^BY^ *MSS11*^3S^ *SFL1*^BY^. Using PCR, an amplicon of the gene of interest was tailed at the 3’ end with the 5’ end of the *kanMX* cassette, and an amplicon of *kanMX* was tailed on the 3’ end with the region immediately downstream of the gene [26]. The two PCR products were co-transformed into a given strain using the lithium acetate method [65] and plated on YPD agar containing G418 to screen for successful integration. Colonies that showed G418 resistance were then checked by PCR and Sanger sequencing to ensure they harbored the allele replacement. To clone the causal nucleotide in *MSS11*, adaptamer-mediated allele replacement was performed multiple times using *MSS11* amplicons that spanned only a part of the gene’s coding region. These specific engineerings are shown in more detail in **S6 Fig**. Each of these engineerings to clone the causal variant in *MSS11* were checked by Sanger sequencing. The gene deletions described in **S4 Note** were performed by replacing a gene of interest with the CORE cassette [64]. Regions corresponding to 60 bases upstream and downstream of the target gene were tailed to the CORE cassette using PCR. This product was transformed into cells using the lithium acetate method [65], and selection with G418 was used to screen for integration of the cassette. PCR was then used to verify that deletion strains recovered from the G418 selection lacked the gene of interest. All primers used for genetic engineering are described in **S5 Table**.

## Supporting Information

S1 Fig Bulk segregant mapping results for each temperature sensitivity class. Genome-wide allele frequencies are presented for the (**A**) HS, (**B**) MS, and (**C**) NS mapping populations. For each of the three plots, allele frequencies in the BY and 3S backcrosses are depicted in the top and bottom panels, respectively. Approximately half of the genome segregates in each backcross and the regions that segregate in one backcross are fixed in the other. Loci that differ significantly from the expected frequency of 0.5 (Materials and Methods) are labeled with highlighted bars: significantly enriched loci from the BY and 3S parents are highlighted in blue and orange, respectively. Two selectable markers on Chromosomes III and V correspond to *MAT****a*** and *can1Δ*::*STE2-SpHIS5*, respectively, and are highlighted in grey (Materials and Methods). The *ira2Δ*2933 allele was a spontaneous mutation that occurred on the 3S chromosome of a BY/3S diploid and is highlighted in red. The results shown in this figure are summarized in the main text in **Fig 2B**.
(TIF)

S2 Fig Two multi-locus genotypes express the rough phenotype exclusively at 21 and 30°C. Only alleles involved in the *END3*^BY^ *FLO8*^3S^ *ira2*Δ2933 *MSS11*^BY^ *TRR1*^3S^ genotype were detected in the bulk segregant mapping data for the MS class. However, we have previously shown that the *END3*^3S^ *FLO8*^3S^ *ira2*Δ2933 *MGA1*^BY^ *MSS11*^BY^ *SFL1*^BY^ genotype can also lead to expression of the rough phenotype at 30°C. Here, we provide examples of strains carrying the (**A**) *END3*^BY^ *FLO8*^3S^ *ira2*Δ2933 *MSS11*^BY^ *TRR1*^3S^ and (**B**) *END3*^3S^ *FLO8*^3S^ *ira2*Δ2933 *MGA1*^BY^ *MSS11*^BY^ *SFL1*^BY^ genotypes. Both of these strains express the rough phenotype at 21 and 30°C, but not 37°C.
(TIF)

S3 Fig Genome-wide scan for pairs of loci that show correlated allele states among sequenced individuals from the HS class. We performed χ^2^ tests on all possible pairs of segregating genomic segments in the (**A**) BY and (**B**) 3S backcross populations. No locus pairs were detected in the BY backcross, while two pairs of loci were detected in the 3S backcross. One of these pairs corresponds to *MSS11* and *SFL1*, while the other corresponds to *MSS11* and a new locus on Chromosome XII that was not identified in our past work.
(TIF)

S4 Fig Multiple alleles are required for rough morphology in a *MSS11*^3S^ HS individual. Here, we performed allele replacements to verify that *END3*^BY^, *MGA1*^BY^, and *SFL1*^BY^ play causal roles in enabling the XII^3S^ *END3*^BY^ *FLO8*^3S^ *ira2*Δ2933 *MGA1*^BY^ *MSS11*^3S^ *SFL1*^BY^ genotype to express the rough phenotype exclusively at 21°C. We used genetic engineering to swap the BY allele of *END3*, *MGA1*, or *SFL1* with the 3S allele in a segregant carrying the aforementioned *MSS11*^3S^-dependent HS genotype (Materials and Methods). Each allele swap resulted in loss of the rough phenotype at 21°C.
(TIF)

S5 Fig *MGA1*^BY^ is required for trait expression at 37°C in an NS genetic background. We replaced *MGA1*^BY^ with *MGA1*^3S^ in an *END3*^BY^ *FLO8*^3S^ *FLO11*^3S^ *ira2Δ*2933 *MGA1*^BY^ *MSS11*^BY^ *SFL1*^BY^ *TRR1*^3S^ genetic background. This allele replacement resulted in a conversion from rough to smooth colony morphology specifically at 37°C.
(TIF)

S6 Fig Cloning of the causal variant in *MSS11*. (**A**) We fine-mapped the causal nucleotide in *MSS11* by performing multiple genetic engineerings in which only part of the gene was replaced in an *END3*^3S^ *FLO8*^3S^ *ira2*Δ2933 *MGA1*^BY^ *MSS11*^BY^ *SFL1*^BY^ genetic background, as indicated by the portion of the gene shown in orange. Vertical bars indicate the locations of SNPs differentiating BY and 3S. The causal SNP is denoted by a black triangle and a scale bar is provided in base pairs. (**B**) The causal nucleotide in *MSS11* results in an isoleucine to serine amino acid substitution in the LisH domain required for Flo8-Mss11 dimerization. 3S carries the derived, serine allele of this amino acid. Inspection of the *MSS11* genotypes of other sequenced *S*. *cerevisiae* isolates revealed that roughly 56% of strains also harbor the serine allele. Mss11 protein sequence data were obtained from the *Saccharomyces* Genome Database (http://www.yeastgenome.org). A scale bar is provided in amino acids.
(TIF)

S1 Note The *ira2Δ*2933 allele was highly enriched (86.1% frequency) but not completely fixed among HS individuals in the BY backcross. Based on whole genome sequencing described later in the paper, we identified 16 *IRA2*^BY^ individuals. These individuals were excluded from further consideration, as our goal in this paper was to characterize GxE in an *ira2Δ*2933 background. We also note that the number of these individuals was too low to enable detection of loci that enable rough morphology in the absence of *ira2Δ*2933.
(DOCX)

S2 Note Because the *END3*^BY^- and *END3*^3S^-dependent genotypes require five and six alleles, respectively, the latter genotype is expected to only occur half as often. In practice, the bias towards the *END3*^BY^-dependent genotype is typically even higher. This is because a locus that confers a selective advantage during random spore isolation in the BYx3S cross is closely linked to *END3*, with the BY allele of this locus conferring a benefit [10,11].
(DOCX)

S3 Note Nineteen MS individuals from the 3S backcross population were randomly chosen, and genotyped at *END3* and *MGA1*. Fourteen of these individuals carried *END3*^BY^, and the BY and 3S alleles of *MGA1* were present in equal frequencies among these *END3*^BY^ MS individuals. In contrast, all five segregants carrying *END3*^3S^ also harbored *MGA1*^BY^. These are consistent with our past results that two genotypes—*END3*^BY^ *FLO8*^3S^ *ira2*Δ2933 *MSS11*^BY^ *TRR1*^3S^ and *END3*^3S^ *FLO8*^3S^ *ira2*Δ2933 *MGA1*^BY^ *MSS11*^BY^ *SFL1*^BY^—underlie the MS class, as *MGA1*^BY^ co-segregates with *END3*^3S^ but exhibits no such association with *END3*^BY^.
(DOCX)

S4 Note The Chromosome XII interval was delimited to a 34,519 base region. Within this interval, the candidate genes *HAP1*, *HSP60*, *GSY2*, *LCB5*, *PDR8*, *SYM1*, *YLR257W*, and *YPT6* were independently deleted in a 3S backcross segregant expressing the HS phenotype and carrying the XII^3S^ *END3*^BY^ *FLO8*^3S^ *ira2*Δ2933 *MGA1*^BY^ *MSS11*^3S^ *SFL1*^BY^ genotype (Materials and Methods). None of these gene deletions resulted in a loss of rough morphology. This indicates that either the causal allele at XII^3S^ is a loss-of-function polymorphism or none of the tested genes are the causal gene at this locus.
(DOCX)

S1 Table Phenotypes of BYx3S *ira2Δ*2933 backcross segregants in preliminary and secondary screens. (XLSX)

S2 Table Initial screen for rough morphology among segregants isolated at three different temperatures. Random spore plates from the BY backcross were screened for rough colonies at 21, 30, or 37°C (Materials and Methods). Rough individuals isolated from each of the temperatures were then examined at all three temperatures for the ability to express the phenotype.
(DOCX)

S3 Table Classification of BY backcross segregants obtained from the preliminary screen into the three temperature sensitivity classes. These data are based on the same individuals and phenotyping results described in **S2 Table**.
(DOCX)

S4 Table Bulk segregant mapping populations were generated for each temperature sensitivity class. Subsets of segregants from each backcross and sensitivity class were gathered for pooled sequencing. Of the 1,010 collected segregants at 21°C, 78.4% of them exhibited the HS, MS, or NS phenotype. DNA from between 51 and 131 individuals of each backcross and class was combined to form six pools. Each pool was sequenced to a minimum coverage of 114X.
(DOCX)

S5 Table Primers used in this study. (XLSX)
