# Supplementary figures and images for: Multi-locus Genotypes Underlying Temperature Sensitivity in a Mutationally Induced Trait
Source: PLoS Genet. 2016 Mar 18;12(3):e1005929. doi: 10.1371/journal.pgen.1005929 (PMC4798298; doi:10.1371/journal.pgen.1005929)

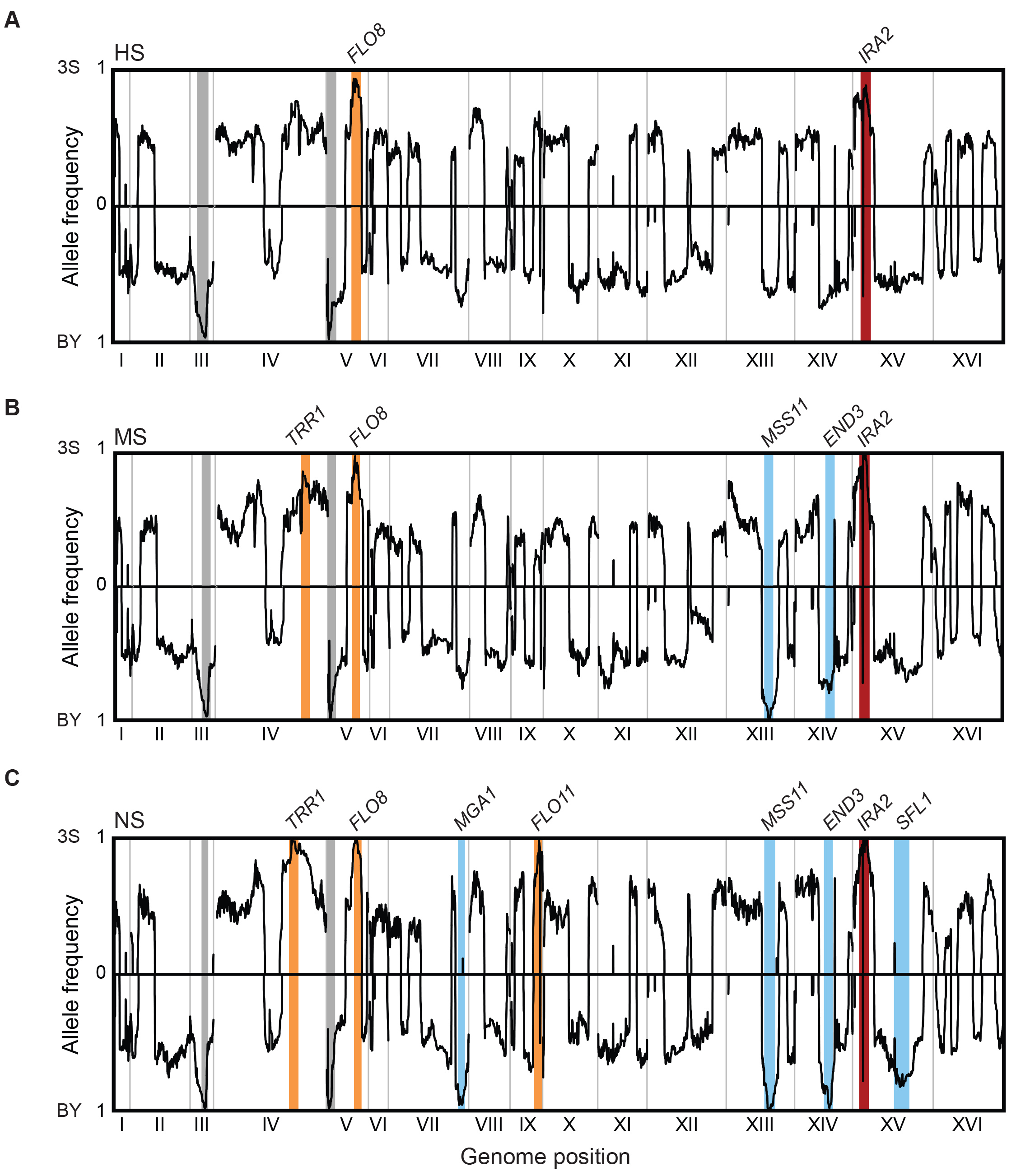

Supplement: S1 Fig — Genome-wide allele frequencies are presented for the (A) HS, (B) MS, and (C) NS mapping populations. For each of the three plots, allele frequencies in the BY and 3S backcrosses are depicted in the top and bottom panels, respectively. Approximately half of the genome segregates in each backcross and the regions that segregate in one backcross are fixed in the other. Loci that differ significantly from the expected frequency of 0.5 (Materials and Methods) are labeled with highlighted bars: significantly enriched loci from the BY and 3S parents are highlighted in blue and orange, respectively. Two selectable markers on Chromosomes III and V correspond to MATa and can1Δ::STE2-SpHIS5, respectively, and are highlighted in grey (Materials and Methods). The ira2Δ2933 allele was a spontaneous mutation that occurred on the 3S chromosome of a BY/3S diploid and is highlighted in red. The results shown in this figure are summarized in the main text in Fig 2B. (TIF) [file pgen.1005929.s001.tif]

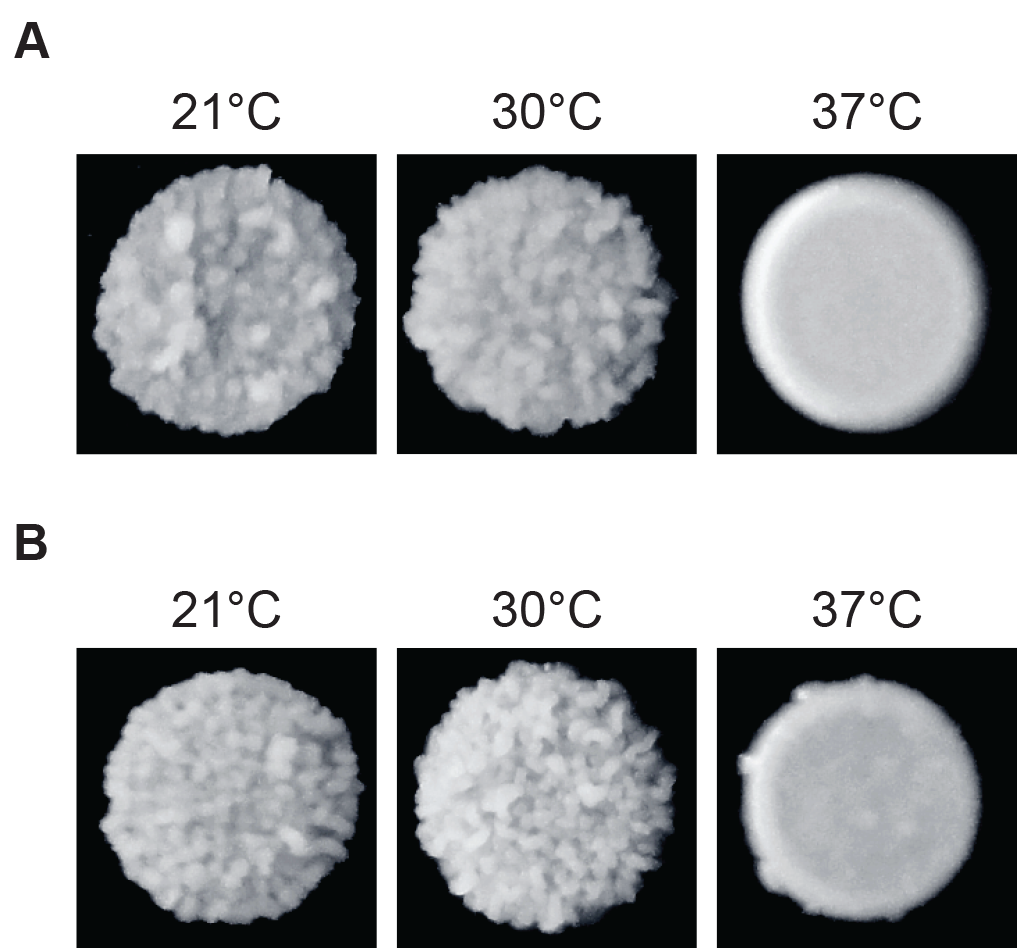

Supplement: S2 Fig — Only alleles involved in the END3BY FLO83S ira2Δ2933 MSS11BY TRR13S genotype were detected in the bulk segregant mapping data for the MS class. However, we have previously shown that the END33S FLO83S ira2Δ2933 MGA1BY MSS11BY SFL1BY genotype can also lead to expression of the rough phenotype at 30°C. Here, we provide examples of strains carrying the (A) END3BY FLO83S ira2Δ2933 MSS11BY TRR13S and (B) END33S FLO83S ira2Δ2933 MGA1BY MSS11BY SFL1BY genotypes. Both of these strains express the rough phenotype at 21 and 30°C, but not 37°C. (TIF) [file pgen.1005929.s002.tif]

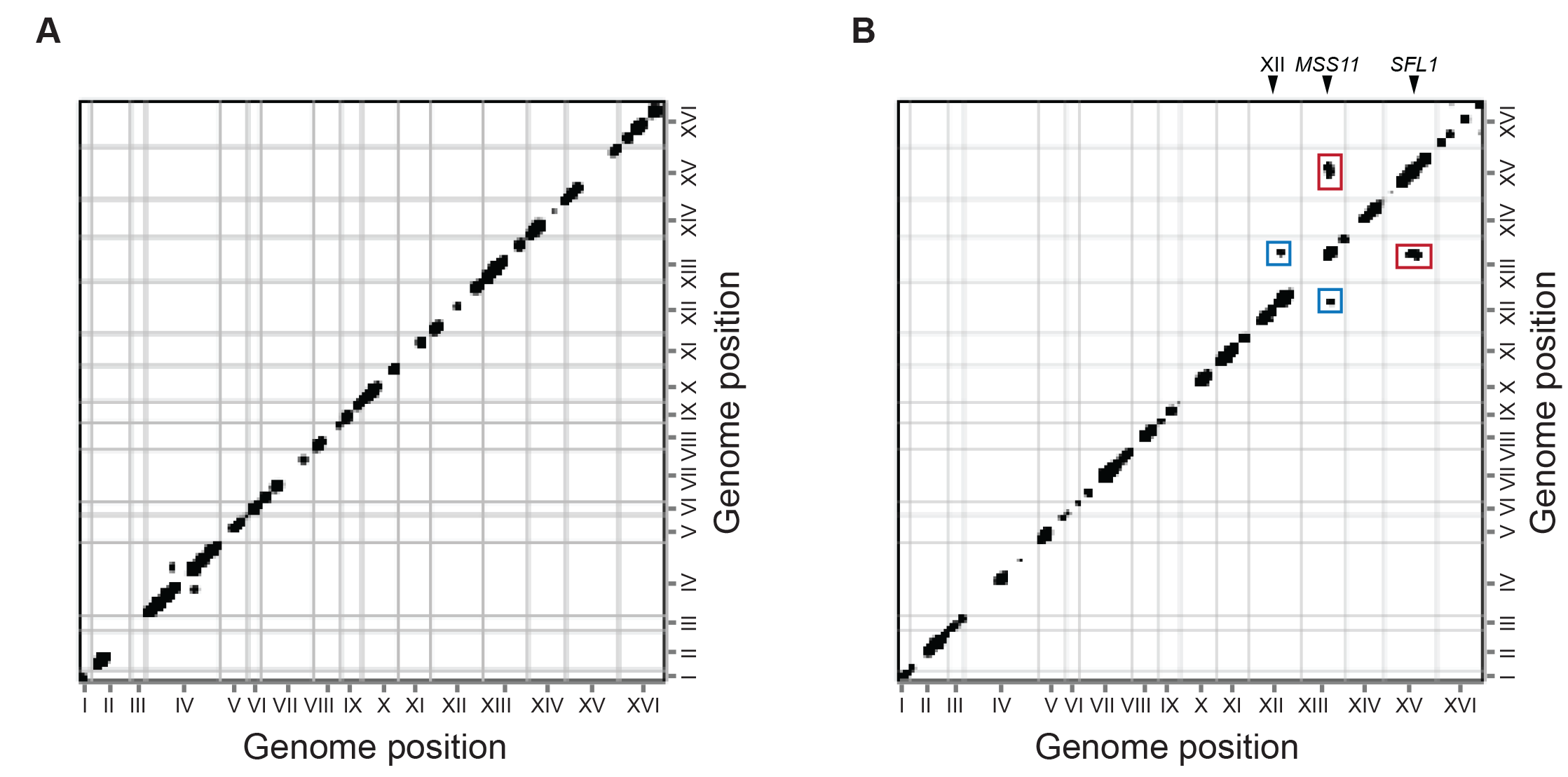

Supplement: S3 Fig — We performed χ2 tests on all possible pairs of segregating genomic segments in the (A) BY and (B) 3S backcross populations. No locus pairs were detected in the BY backcross, while two pairs of loci were detected in the 3S backcross. One of these pairs corresponds to MSS11 and SFL1, while the other corresponds to MSS11 and a new locus on Chromosome XII that was not identified in our past work. (TIF) [file pgen.1005929.s003.tif]

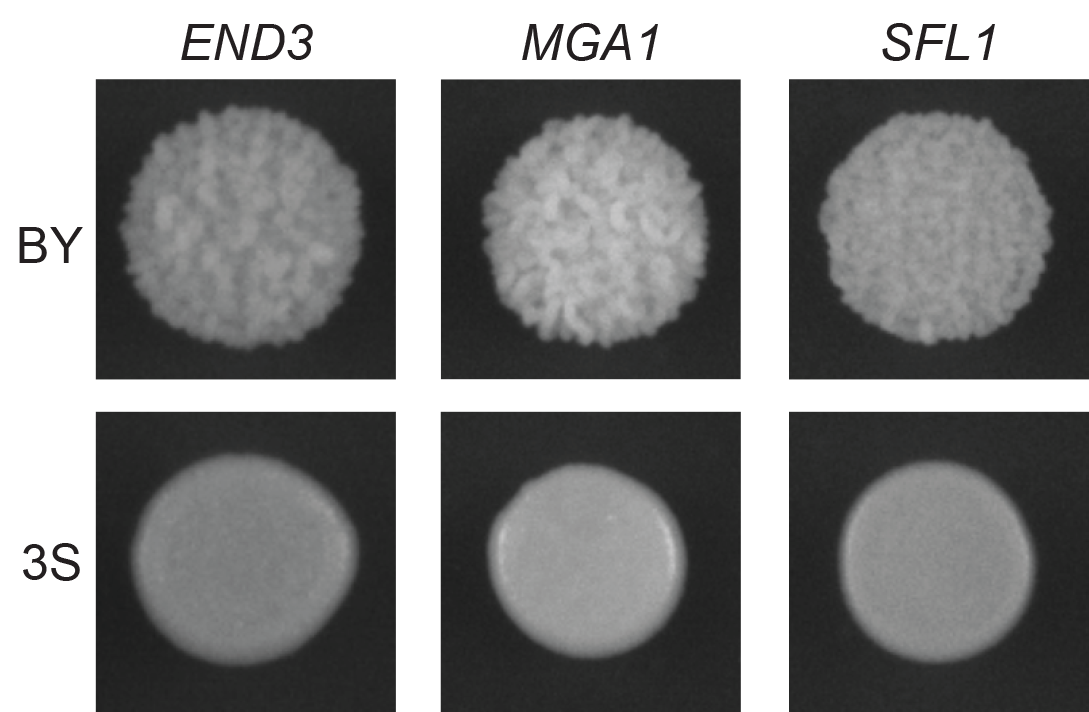

Supplement: S4 Fig — Here, we performed allele replacements to verify that END3BY, MGA1BY, and SFL1BY play causal roles in enabling the XII3S END3BY FLO83S ira2Δ2933 MGA1BY MSS113S SFL1BY genotype to express the rough phenotype exclusively at 21°C. We used genetic engineering to swap the BY allele of END3, MGA1, or SFL1 with the 3S allele in a segregant carrying the aforementioned MSS113S-dependent HS genotype (Materials and Methods). Each allele swap resulted in loss of the rough phenotype at 21°C. (TIF) [file pgen.1005929.s004.tif]

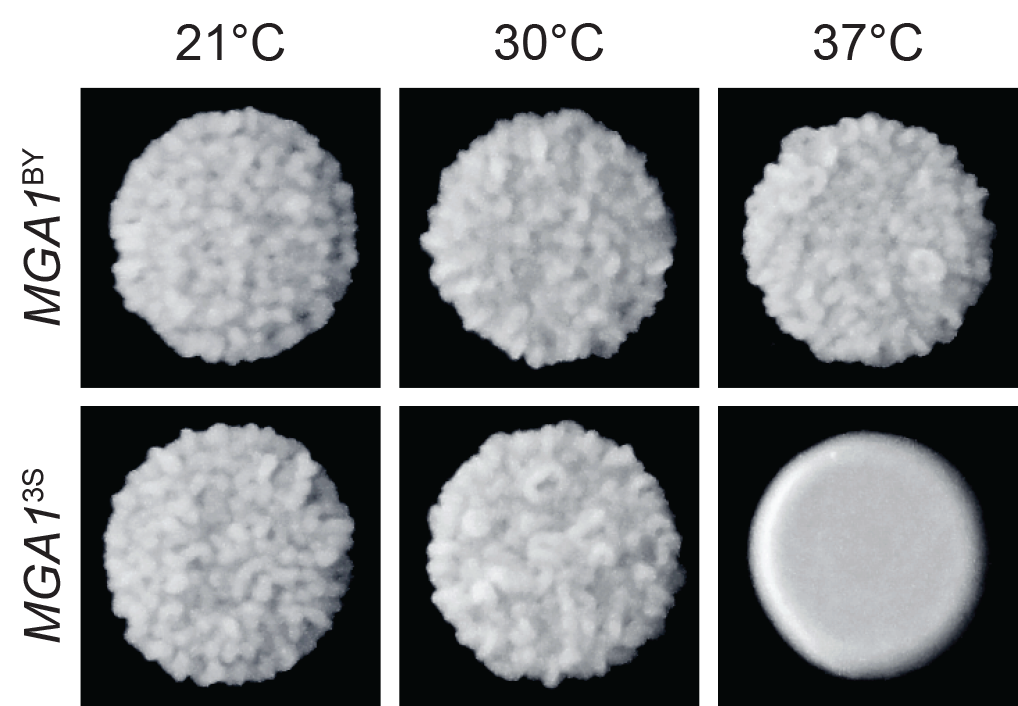

Supplement: S5 Fig — We replaced MGA1BY with MGA13S in an END3BY FLO83S FLO113S ira2Δ2933 MGA1BY MSS11BY SFL1BY TRR13S genetic background. This allele replacement resulted in a conversion from rough to smooth colony morphology specifically at 37°C. (TIF) [file pgen.1005929.s005.tif]

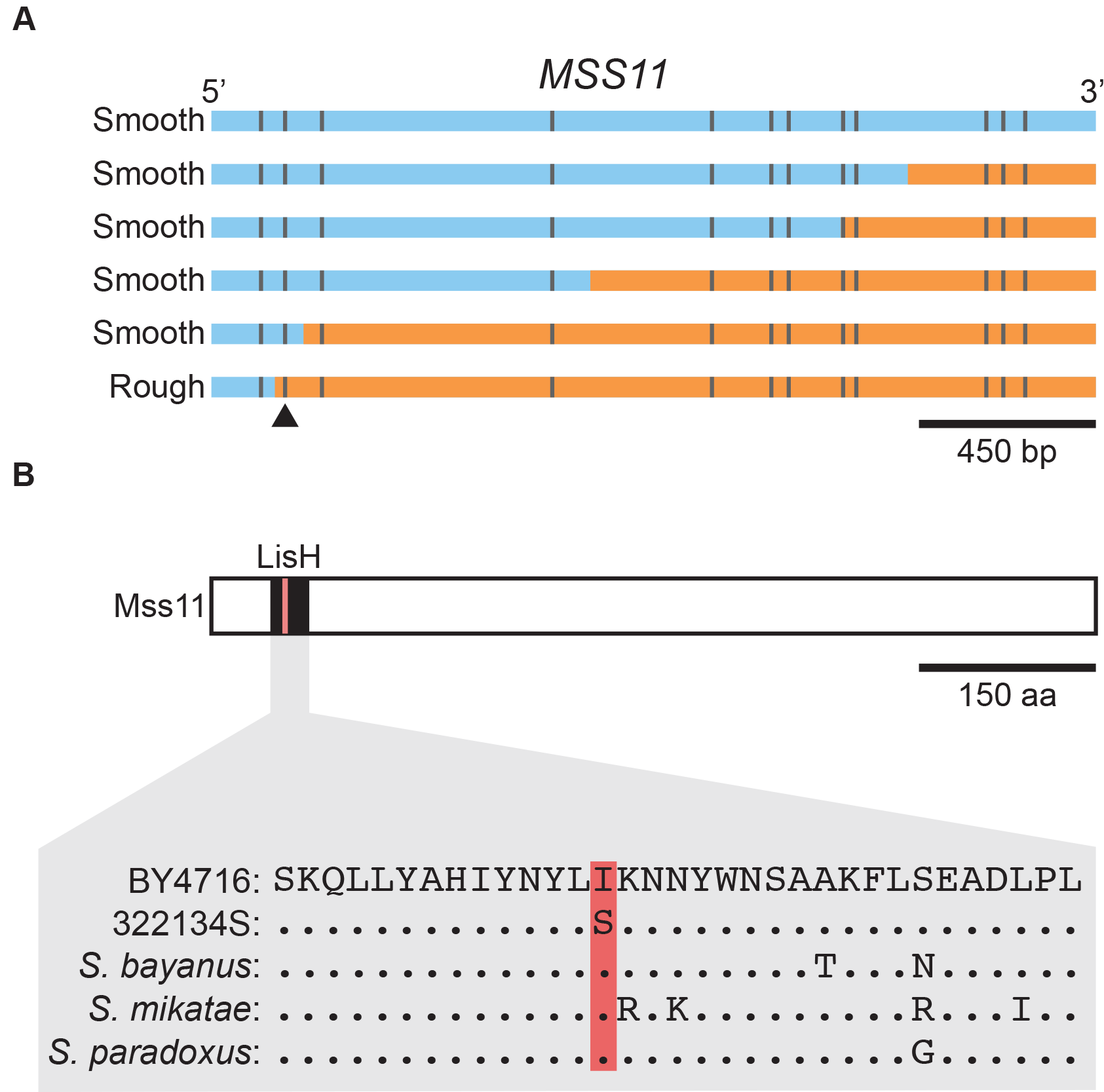

Supplement: S6 Fig — (A) We fine-mapped the causal nucleotide in MSS11 by performing multiple genetic engineerings in which only part of the gene was replaced in an END33S FLO83S ira2Δ2933 MGA1BY MSS11BY SFL1BY genetic background, as indicated by the portion of the gene shown in orange. Vertical bars indicate the locations of SNPs differentiating BY and 3S. The causal SNP is denoted by a black triangle and a scale bar is provided in base pairs. (B) The causal nucleotide in MSS11 results in an isoleucine to serine amino acid substitution in the LisH domain required for Flo8-Mss11 dimerization. 3S carries the derived, serine allele of this amino acid. Inspection of the MSS11 genotypes of other sequenced S. cerevisiae isolates revealed that roughly 56% of strains also harbor the serine allele. Mss11 protein sequence data were obtained from the Saccharomyces Genome Database (http://www.yeastgenome.org). A scale bar is provided in amino acids. (TIF) [file pgen.1005929.s006.tif]
