# Supplementary material for: Multi-locus Genotypes Underlying Temperature Sensitivity in a Mutationally Induced Trait
Source: PLoS Genet. 2016 Mar 18;12(3):e1005929. doi: 10.1371/journal.pgen.1005929 (PMC4798298; doi:10.1371/journal.pgen.1005929)
Supplement: S1 Note — Based on whole genome sequencing described later in the paper, we identified 16 IRA2BY individuals. These individuals were excluded from further consideration, as our goal in this paper was to characterize GxE in an ira2Δ2933 background. We also note that the number of these individuals was too low to enable detection of loci that enable rough morphology in the absence of ira2Δ2933. (DOCX) [file pgen.1005929.s007.docx]

**S1 Note.** The *ira2∆*2933 allele was highly enriched (86.1% frequency) but not completely fixed among HS individuals in the BY backcross. Based on whole genome sequencing described later in the paper, we identified 16 *IRA2*^BY^ individuals. These individuals were excluded from further consideration, as our goal in this paper was to characterize GxE in an *ira2∆*2933 background. We also note that the number of these individuals was too low to enable detection of loci that enable rough morphology in the absence of *ira2∆*2933.
