# Supplementary material for: Multi-locus Genotypes Underlying Temperature Sensitivity in a Mutationally Induced Trait
Source: PLoS Genet. 2016 Mar 18;12(3):e1005929. doi: 10.1371/journal.pgen.1005929 (PMC4798298; doi:10.1371/journal.pgen.1005929)
Supplement: S2 Note — In practice, the bias towards the END3BY-dependent genotype is typically even higher. This is because a locus that confers a selective advantage during random spore isolation in the BYx3S cross is closely linked to END3, with the BY allele of this locus conferring a benefit [10,11]. (DOCX) [file pgen.1005929.s008.docx]

**S2 Note.** Because the *END3*^BY^- and *END3*^3S^-dependent genotypes (**Fig. 4A**) require five and six alleles, respectively, the latter genotype is expected to only occur half as often. In practice, the bias towards the *END3*^BY^-dependent genotype is typically even higher. This is because a locus that confers a selective advantage during random spore isolation in the BYx3S cross is closely linked to *END3*, with the BY allele of this locus conferring a benefit (see Taylor and Ehrenreich. 2014. PLOS Genetics; Taylor and Ehrenreich. 2015. PLOS Genetics).
