# Supplementary material for: Multi-locus Genotypes Underlying Temperature Sensitivity in a Mutationally Induced Trait
Source: PLoS Genet. 2016 Mar 18;12(3):e1005929. doi: 10.1371/journal.pgen.1005929 (PMC4798298; doi:10.1371/journal.pgen.1005929)
Supplement: S3 Note — Fourteen of these individuals carried END3BY, and the BY and 3S alleles of MGA1 were present in equal frequencies among these END3BY MS individuals. In contrast, all five segregants carrying END33S also harbored MGA1BY. These are consistent with our past results that two genotypes—END3BY FLO83S ira2Δ2933 MSS11BY TRR13S and END33S FLO83S ira2Δ2933 MGA1BY MSS11BY SFL1BY—underlie the MS class, as MGA1BY co-segregates with END33S but exhibits no such association with END3BY. (DOCX) [file pgen.1005929.s009.docx]

**S3 Note.** 19 MS individuals from the 3S backcross population were randomly chosen, and genotyped at *END3* and *MGA1*. 14 of these individuals carried *END3*^BY^, and the BY and 3S alleles of *MGA1* were present in equal frequencies among these *END3*^BY^ MS individuals. In contrast, all five segregants carrying *END3*^3S^ also harbored *MGA1*^BY^. These are consistent with our past results that two genotypes—*END3*^BY^ *FLO8*^3S^ *ira2*∆2933 *MSS11*^BY^ *TRR1*^3S^ and *END3*^3S^ *FLO8*^3S^ *ira2*∆2933 *MGA1*^BY^ *MSS11*^BY^ *SFL1*^BY^—underlie the MS class, as *MGA1*^BY^ co-segregates with *END3*^3S^ but exhibits no such association with *END3*^BY^.
