# Supplementary material for: Multi-locus Genotypes Underlying Temperature Sensitivity in a Mutationally Induced Trait
Source: PLoS Genet. 2016 Mar 18;12(3):e1005929. doi: 10.1371/journal.pgen.1005929 (PMC4798298; doi:10.1371/journal.pgen.1005929)
Supplement: S4 Note — Within this interval, the candidate genes HAP1, HSP60, GSY2, LCB5, PDR8, SYM1, YLR257W, and YPT6 were independently deleted in a 3S backcross segregant expressing the HS phenotype and carrying the XII3S END3BY FLO83S ira2Δ2933 MGA1BY MSS113S SFL1BY genotype (Materials and Methods). None of these gene deletions resulted in a loss of rough morphology. This indicates that either the causal allele at XII3S is a loss-of-function polymorphism or none of the tested genes are the causal gene at this locus. (DOCX) [file pgen.1005929.s010.docx]

**S4 Note.** The Chromosome XII interval was delimited to a 34,519 base region. Within this interval, the candidate genes *HAP1*, *HSP60*, *GSY2*, *LCB5*, *PDR8*, *SYM1*, *YLR257W*, and *YPT6* were independently deleted in a 3S backcross segregant expressing the HS phenotype and carrying the XII^3S^ *END3*^BY^ *FLO8*^3S^ *ira2*∆2933 *MGA1*^BY^ *MSS11*^3S^ *SFL1*^BY^ genotype (**Methods**). None of these gene deletions resulted in a loss of rough morphology. This indicates that either the causal allele at XII^3S^ is a loss-of-function polymorphism or none of the tested genes are the causal gene at this locus.
