# Supplementary material for: Multi-locus Genotypes Underlying Temperature Sensitivity in a Mutationally Induced Trait
Source: PLoS Genet. 2016 Mar 18;12(3):e1005929. doi: 10.1371/journal.pgen.1005929 (PMC4798298; doi:10.1371/journal.pgen.1005929)
Supplement: S2 Table — Random spore plates from the BY backcross were screened for rough colonies at 21, 30, or 37°C (Materials and Methods). Rough individuals isolated from each of the temperatures were then examined at all three temperatures for the ability to express the phenotype. (DOCX) [file pgen.1005929.s012.docx]

| Collection  Temperature  (°C) | Rough  Segregants  Collected | Rough at 21°C | Rough at  30°C | Rough at  37°C |
| --- | --- | --- | --- | --- |
| 21 | 173 | 173 | 66 | 56 |
| 30 | 107 | 102 | 107 | 49 |
| 37 | 72 | 68 | 47 | 72 |

**S2 Table. Initial screen for rough morphology among segregants isolated at three different temperatures.** Random spore plates from the BY backcross were screened for rough colonies at 21, 30, or 37°C (**Methods**). Rough individuals isolated from each of the temperatures were then examined at all three temperatures for the ability to express the phenotype.
