# Supplementary material for: Multi-locus Genotypes Underlying Temperature Sensitivity in a Mutationally Induced Trait
Source: PLoS Genet. 2016 Mar 18;12(3):e1005929. doi: 10.1371/journal.pgen.1005929 (PMC4798298; doi:10.1371/journal.pgen.1005929)
Supplement: S3 Table — These data are based on the same individuals and phenotyping results described in S2 Table. (DOCX) [file pgen.1005929.s013.docx]

| Collection  Temperature (°C) | Rough  Segregants  Collected | Number  of HS  Segregants | Number  of MS  Segregants | Number  of NS  Segregants | % Segregants  in HS, MS, or  NS classes |
| --- | --- | --- | --- | --- | --- |
| 21 | 173 | 43 | 27 | 31 | 60.7 |
| 30 | 107 |  | 40 | 48 | 91.6 |
| 37 | 72 |  |  | 47 | 65.3 |

**S3 Table. Classification of BY backcross segregants obtained from the preliminary screen into the three temperature sensitivity classes.** These data are based on the same individuals and phenotyping results described in **S2 Table**.
