# Supplementary material for: Multi-locus Genotypes Underlying Temperature Sensitivity in a Mutationally Induced Trait
Source: PLoS Genet. 2016 Mar 18;12(3):e1005929. doi: 10.1371/journal.pgen.1005929 (PMC4798298; doi:10.1371/journal.pgen.1005929)
Supplement: S4 Table — Subsets of segregants from each backcross and sensitivity class were gathered for pooled sequencing. Of the 1,010 collected segregants at 21°C, 78.4% of them exhibited the HS, MS, or NS phenotype. DNA from between 51 and 131 individuals of each backcross and class was combined to form six pools. Each pool was sequenced to a minimum coverage of 114X. (DOCX) [file pgen.1005929.s014.docx]

| Sensitivity Class | Collected | | Sequenced | | Coverage | |
| --- | --- | --- | --- | --- | --- | --- |
|  | BY | 3S | BY | 3S | BY | 3S |
| **HS** | 257 | 217 | 82 | 131 | 342.88 | 378.46 |
| **MS** | 74 | 139 | 55 | 101 | 209.63 | 406.09 |
| **NS** | 53 | 52 | 51 | 51 | 114.79 | 397.37 |
| **Other** | 160 | 58 | 0 | 0 | NA | NA |

**S4 Table. Bulk segregant mapping populations were generated for each temperature sensitivity class.** Subsets of segregants from each backcross and sensitivity class were gathered for pooled sequencing. Of the 1,010 collected segregants at 21°C, 78.4% of them exhibited the HS, MS, or NS phenotype. DNA from between 51 and 131 individuals of each backcross and class was combined to form six pools. Each pool was sequenced to a minimum coverage of 114X.
